# Pituitary hyperplasia secondary to acquired hypothyroidism: case report

**DOI:** 10.1186/1824-7288-37-15

**Published:** 2011-04-07

**Authors:** Roberto Franceschi, Umberto Rozzanigo, Riccarda Failo, Maria Bellizzi, Annunziata Di Palma

**Affiliations:** 1 Pediatrics Unit, S.Chiara Hospital of Trento, Largo Medaglie d'Oro 9, 38122 Trento, Italy; 2 Diagnostic Imaging Unit, S.Chiara Hospital of Trento, Largo Medaglie d'Oro 9, 38122 Trento, Italy

## Abstract

**Objective and Importance:**

despite recent progress in imaging, it is still difficult to distinguish between pituitary adenoma and hyperplasia, even using Magnetic Resonance Imaging (MRI) with gadolinium injection. We describe an example of reactive pituitary hyperplasia from primary hypothyroidism that mimicked a pituitary macroadenoma in a child.

**Clinical Presentation:**

a 10 year old boy presented with headache and statural growth arrest. MRI revealed an intrasellar and suprasellar pituitary mass. Endocrine evaluation revealed primary hypothyroidism.

**Intervention:**

the patient was started on levothyroxine with resolution of the mass effect.

**Conclusion:**

primary hypothyroidism should be considered in the differential diagnosis of solid mass lesions of the pituitary gland. Examination of thyroid function in patients with sellar and suprasellar masses revealed by MRI may avoid unnecessary operations which can cause irreversible complications.

## Background

There are many causes of sellar and suprasellar mass, and pituitary enlargment secondary to primary hypothyroidism has been previously described [1-5]. It results from the loss of thyroxine feedback inhibition and the subsequent overproduction of thyrotropin-releasing hormone (TRH) [6]. Despite recent progress in imaging techniques, it is not possible to distinguish between TSH-producing macroadenoma and hyperplasia of pituitary thyrotroph cells on CT and MR scans. In such cases, repeat MRI after therapy with thyroxine may provide a definitive diagnosis and eliminate unnecessary surgery: unlike adenoma, pituitary hyperplasia resolves after thyroid hormone replacement therapy [2].

## Case Presentation

### History and Physical Examination

A 10 year old boy, born at term in Morocco from non-consanguineous parents after an uncomplicated pregnancy, presented at his local hospital with occipital headache over the last three months and height growth arrest: during the previous 2 years he had grown 1 cm, declining from the 75-90^th ^percentile to the 25^th^, while his weight had passed from the 50^th ^to the 75^th ^percentile in the last year. He did not complain of vomiting, tiredness or any other symptoms.

His past and familial history was unremarkable and his cognitive development normal. On physical examination, he presented with height 136 cm (25^th ^percentile), weight 42 kg (75^th ^percentile), prepubertal status, blood pressure 120/67 mmHg, pulse rate 72 bpm, and non-palpable thyroid gland. Because of persistent headache he was referred to the neurologist at his local hospital and the examination was normal, as were the *fundus oculi *and visual fields.

### Radiographic and laboratory data

**Figure 1:**
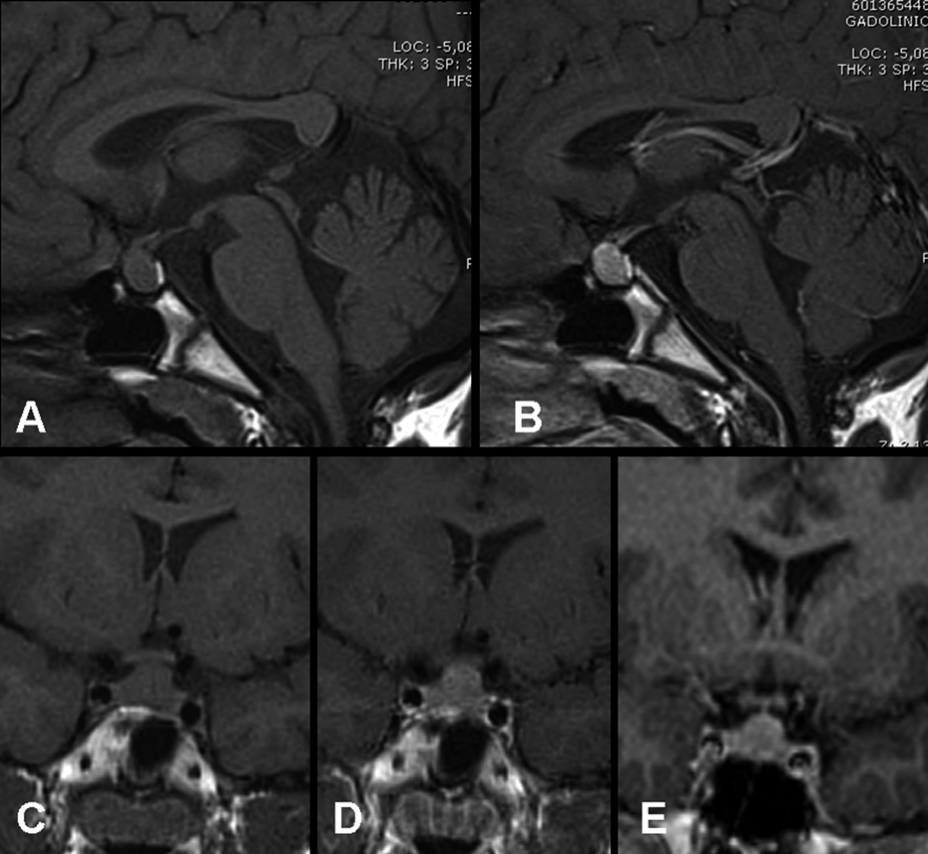
**A-E. Pretreatment sagittal (A, B) and coronal (C-E) T1-weighted MR images show diffuse enlagement of the pituitary gland, that extends into the suprasellar cystern with mild compression of the optic chiasm and of the posterior neurohypophyseal lobe, which keeps its normal bright signal**. After intravenous Gadolinum administration (B, D) the mass demonstrate homogeneous enhancement; the pituitary stalk (E) is raised but still along the midline.

Cranial Magnetic Resonance Imaging (MRI) was performed to rule out an organic lesion and an intrasellar and suprasellar pituitary mass was detected. The enlargment was isointense to gray matter, extended on the suprasellar cistern with mild compression of the optic chiasm. The pituitary stalk and posterior pituitary were dislocated. After gadolinium the mass homogeneously enhanced (Figure 1).

After endocrine consultation, blood tests were done: full blood count, erythrocyte sedimentation rate, renal and liver function and anti-coeliac screening were

**Figure 2:**
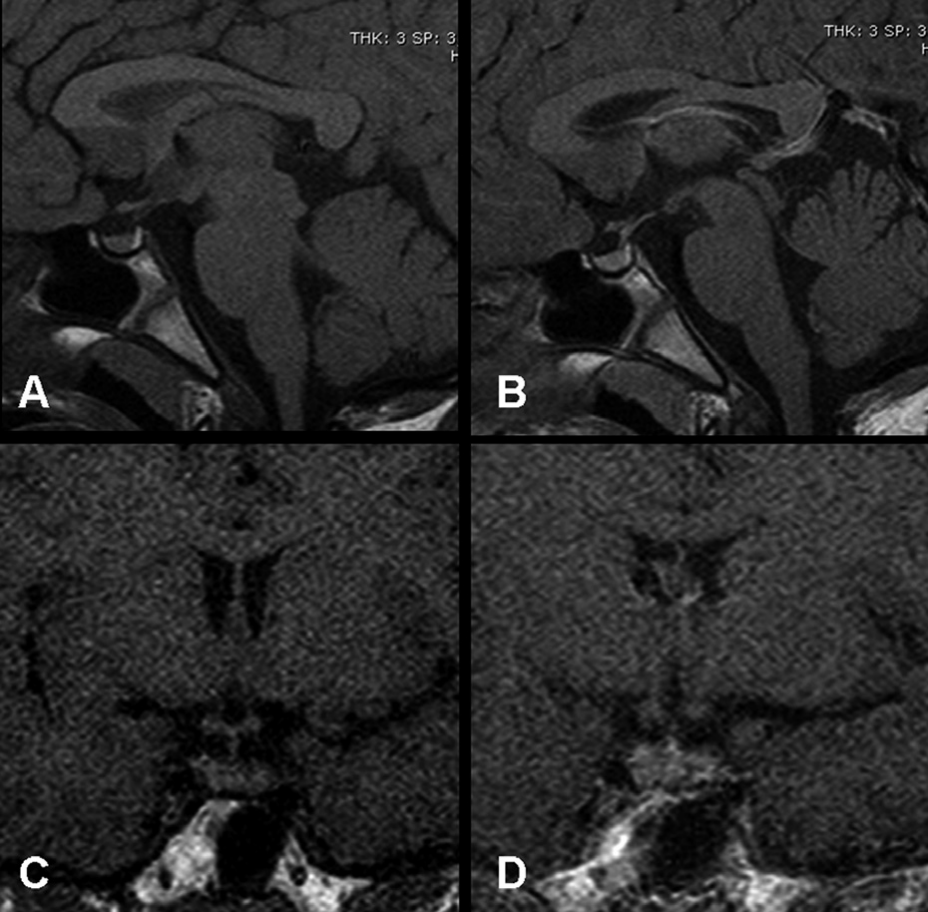
**A-D. After 5 months of thyroid hormone replacement therapy sagittal (A, B) and coronal (C-D) MR images without (A, C) and with (B-D) Gadolinium administration reveal decrease in size of the pituitary gland and regression of the mass effect**.

Thyroid ultrasound revealed normal thyroid size, with markedly heterogeneous echo texture and hypo-echoic areas. The color Doppler study revealed diffuse hypervascolarization of the gland. The picture was consistent with late-stage chronic thyroiditis. Based on clinical history and laboratory data, a diagnosis of primary hypothyroidism consequent to chronic autoimmune (Hashimoto's) thyroiditis was made and the pituitary mass was attributed to secondary hyperplasia. The patient was started on levothyroxine at 50 ug/day. IGF-I and thyroid function had returned to the normal range after 3 months (Table 1). Height growth velocity increased (7 cm/year) and at the last visit, at 11 years, the boy's height was on the 50^th ^percentile and he was no longer complaining of headache. A follow-up MRI scan 5 months after levothyroxine replacement showed resolution of the mass effect (Figure 2).

**Table 1:** Hormonal pattern at diagnosis and during follow up

Parameter	Before therapy	3 months later	6 months later	Normal range
TSH, mU/L	**589**	**13.8**	4.3	0.20-4.50

Free T4, pmol/L	**1.5**	13.9	14.2	12-22

Free T3, pmol/L	**2.2**	6.2	5.4	3.8-8.6

IGF-I, nmol/L	**8.6**	24.66	n.a.	11.64-34.1

PRL, nmol/L	**1.23**	0.51	n.a.	0.17-0.64

TSH: thyroid-stimulating hormone; T4: thyroxine; T3: triiodothyronine; IGF-I: insulin-like growth factor I; PRL: prolactin. Results are expressed in international unit. For free T4 to convert to ng/dL divide by 12.86; for free T3 to convert to ng/dL divide by 1.536, for IGF-I to convert to ng/mL divide by 0.1307, for PRL to convert to ng/mL divide by 0.0426. N.a.: not available

## Discussion

Due to the loss of thyroxine feedback inhibition and the subsequent overproduction of thyrotropin-releasing hormone (TRH), long-standing hypothyroidism results in hyperplasia of the thyrotroph cells and subsequent enlargement of the pituitary gland [6]. The incidence of pituitary hyperplasia in patients with hypothyroidism varies from 25% to 81% [7], high incidence (70%) is reported in patients with TSH levels ≥50 μIU/m 8 l [8]. Because TRH also has a weak stimulatory effect on lactotroph cells, mild to moderate hyperprolactinemia may also occur in about three-quarters of patients [7]. Nevertheless in our patient the slight increase in prolactin was probably due to the compression of the infundibulum by the mass, rather than to lactotroph hyperplasia.

Children with pituitary hyperplasia and primary hypothyroidism rarely present with neurological symptoms secondary to sellar expansion (3%). Their complaints at presentation are associated with hypothyroidism and abnormal puberty [7]. Most children present with symptoms of growth arrest due to primary hypothyroidism, and growth hormone (GH) production may be decreased because thyroxine is one of the factors stimulating GH synthesis [1]. Long-standing primary hypothyroidism may also lead to irreversible pituitary injury, which can lead to a deficiency in one or more pituitary hormones [3]. Therefore in our patient persistent headache has to be attributed to hypothyroidism, even if the exact mechanism is not clear. Moreover reduced IGF-1 was probably secondary to transitory reduced GH synthesis.

Despite recent progress in imaging, it is still difficult to distinguish between a pituitary adenoma and hyperplasia, even using MRI with gadolinium injection. On imaging studies, secondary pituitary hyperplasia is often characterized by a homogeneously enhancing lesion that may progress rapidly following the onset of a hypothyroid state [9]. Interpretation of a pituitary mass without an endocrine investigation can lead to unnecessary surgery which can cause irreversible complications [9-11]. Differential diagnosis with TSH secreting adenoma can be made by means of inappropriatey normal/high range values of T3 and T4. Nevertheless the sequence and time course of hypothyroidism and pituitary hyperplasia have not been clarified [5]. Concomitant diagnosis of hypothyroidism and pituitary hyperplasia are described in the literature [12], but two additional scenarios have been reported: a) normal thryoid function may be found in patients with a pituitary mass revealed by MR imaging. Re-examination a few weeks later is recommended because initial normal thyroid function may mask the diagnosis of hypothyroidism-related pituitary hyperplasia in these patients [9]; b) in other patients rapid progression of hyperplasia may develop after hypothyroidism [12].

Thyroid hormone replacement therapy led to a decrease in the size of the gland in 85% of patients with pituitary enlargement who underwent follow-up MR examinations [8]. Surgery should be reserved for decompression of the optic chiasm or to obtain a pathological diagnosis in the case of a pituitary mass which does not respond to, or worsens under, thyroid hormone replacement [3]. In our patient, baseline thyroid function tests were consistent with primary hypothyroidism secondary to chronic autoimmune thyroiditis and repeat pituitary MRI after 5 months of levothyroxine therapy revealed complete regression of the pituitary mass. Based on the combined clinical findings, hormonal studies, and repeat MRI findings, the diagnosis of pituitary hyperplasia secondary to primary autoimmune hypothyroidism was made.

## Conclusions

Primary hypothyroidism should be considered in the differential diagnosis of solid mass lesions of the pituitary gland, particularly in children with short stature. Examination of thyroid function in patients with sellar and suprasellar masses revealed by MR imaging may avoid unnecessary operations.

## Competing interests

The authors declare that they have no competing interests.

## Authors' contributions

RF performed the diagnosis and has been involved in drafting the manuscript. UR carried out the MRI. RFA partecipated in the MRI analysis. MB contributed to the patient endocrine follow-up. ADP has been involved in critically revising the manuscript and has given final approval of the version to be published.

All authors read and approved the final manuscript.

## Consent

Written informed consent was obtained from the patient's relatives for publication of this case report and any accompanying images. A copy of the written consent is available for review by the Editor-in-Chief of this journal.
